# Co-regulatory activity of hnRNP K and NS1-BP in influenza and human mRNA splicing

**DOI:** 10.1038/s41467-018-04779-4

**Published:** 2018-06-19

**Authors:** Matthew G. Thompson, Raquel Muñoz-Moreno, Prasanna Bhat, Renat Roytenberg, John Lindberg, Matthew R. Gazzara, Michael J. Mallory, Ke Zhang, Adolfo García-Sastre, Beatriz M. A. Fontoura, Kristen W. Lynch

**Affiliations:** 1 Departments of Biochemistry and Biophysics, Perelman School of Medicine, University of Pennsylvania, 422 Curie Blvd, Philadelphia, PA 19104 USA; 2 Department of Microbiology, Icahn School of Medicine at Mount Sinai, 1468 Madison Ave., Box 1124, New York, NY 10029 USA; 3 Global Health and Emerging Pathogens Institute, Icahn School of Medicine at Mount Sinai, 1468 Madison Ave., Box 1124, New York, NY 10029 USA; 4 Department of Cell Biology, UT Southwestern Medical Center, 6000 Harry Hines Blvd., Dallas, TX 75390 USA; 5 Department of Medicine, Division of Infectious Diseases, Icahn School of Medicine at Mount Sinai, 1468 Madison Ave., Box 1124, New York, NY 10029 USA

## Abstract

Three of the eight RNA segments encoded by the influenza A virus (IAV) undergo alternative splicing to generate distinct proteins. Previously, we found that host proteins hnRNP K and NS1-BP regulate IAV M segment splicing, but the mechanistic details were unknown. Here we show NS1-BP and hnRNP K bind M mRNA downstream of the M2 5′ splice site (5′ss). NS1-BP binds most proximal to the 5′ss, partially overlapping the U1 snRNP binding site, while hnRNP K binds further downstream and promotes U1 snRNP recruitment. Mutation of either or both the hnRNP K and NS1-BP-binding sites results in M segment mis-splicing and attenuated IAV replication. Additionally, we show that hnRNP K and NS1-BP regulate host splicing events and that viral infection causes mis-splicing of some of these transcripts. Therefore, our proposed mechanism of hnRNP K/NS1-BP mediated IAV M splicing provides potential targets of antiviral intervention and reveals novel host functions for these proteins.

## Abstract (web summary)

Alternative splicing of influenza A virus (IAV) M transcript is regulated by hnRNP K and NS1-BP, but mechanistic details are unknown. Here, Thompson et al. show how hnRNP K and NS1-BP bind M mRNA and that these proteins regulate splicing of host transcripts in both the absence and presence of IAV infection.

## Introduction

Viruses share many gene processing steps with human cells and have historically provided critical insight into mammalian processes. Indeed, pre-mRNA splicing was first discovered through the study of viral RNA, and investigation of viral RNA processing continues to reveal mechanistic insight into host mRNA splicing and trafficking.

Influenza A virus (IAV) is an important human pathogen that causes ~250,000 to 500,000 deaths per year worldwide^1^. In pandemic years, influenza infection can lead to even higher mortality rates, as in 1918 when at least 20 million deaths occurred worldwide^2^. Although vaccines and few antiviral drugs are available, both are limited by antigenic drift and shift of the virus as well as the development of resistance^3^. Therefore, it is crucial to understand influenza virus-host interactions in order to identify host vulnerabilities targeted by influenza virus that can reveal new antiviral mechanisms and potentially be used to devise new therapeutic options.

The IAV genome is comprised of 8 single-strand, negative-sense, RNA segments^4^. Three of these segments, M, NS, and PB2 (also called 7, 8 and 1, respectively), undergo alternative splicing^4,5^. Alternative splicing of the M and NS segments produces two essential viral proteins each. For the M segment, the unspliced M1 mRNA encodes the M1 matrix protein located underneath the viral envelope, while intron removal leads to the M2 mRNA, which encodes a proton channel protein (M2) that allows acidification of the virus particle in the endosome/lysosome during viral entry^4^. Other roles of M2 include the promotion of membrane scission during viral budding, the inhibition of autophagic pathways^6,7^ and the recruitment of the host protein Ubr4 to promote optimal surface expression of viral membrane proteins^8^. Thus, an appropriate balance between M1 and M2 mRNAs must be produced in order to achieve an efficient viral infection and replication, and regulation of the splicing of M1 to M2 represents a fundamental step of viral-host interaction that is a potential therapeutic target.

**Fig. 1:**
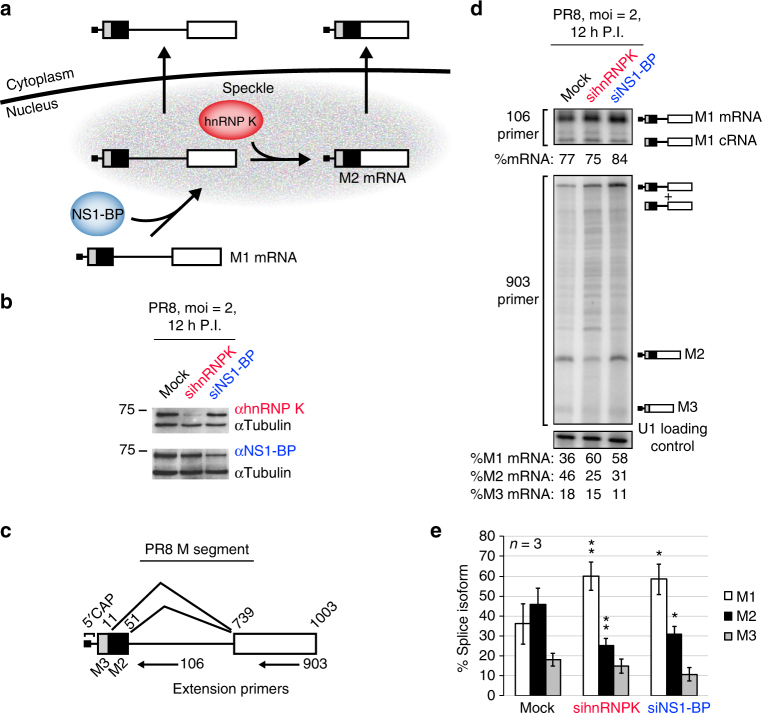
Host proteins hnRNP K and NS1-BP regulate IAV PR8 M segment splicing. **a** Model for hnRNP K and NS1-BP regulated M segment splicing based on previous studies^6,7^. **b** Immunoblots of A549 whole cell extract 12 h post PR8 infection in the context of mock, hnRNP K or NS1-BP siRNA knockdown. Molecular weights are indicated to left of each blot. **c** Diagram of IAV PR8 M segment mRNA with possible splice isoforms and extension primer positions labeled. Boxes denote exons and the line denotes an intron. M3 and M2 indicate potential 5′ss and numbers indicate nucleotide position. **d** Primer extension of PR8 M segment antisense RNA after 12 h infection in A549 cells at moi = 2. Cells were mock transfected or treated with 50 nM siRNA targeted at hnRNP K or NS1-BP. Extension primers used are indicated on the left and band identities on the right of the gel image. Uncropped gels shown in Supplementary Fig. 5. **e** Quantification of mRNA primer extension products represented as percentage signal of each isoform relative to signal of total isoforms (M1 + M2 + M3). Values are means ± s.d. from three independent experiments. For all experiments in manuscript, a minimum of 3 experiments were performed to insure appropriateness of statistical tests. Statistical significance was determined via two-tailed student’s *t* test, where *p < 0.1 and **p < 0.05 when comparing mock vs siRNA samples

Splicing of both viral and host mRNAs is mediated by the spliceosome, a dynamic enzymatic complex composed primarily of 5 ribonucleoprotein particles (U1, U2, U4, U5 U6 snRNPs)^9^. The association of the spliceosome with substrate is typically directed by additional proteins that control the efficiency with which splicing occurs at any given location^10^. We have previously shown that removal of the M segment intron to produce the M2 mRNA is promoted by the host proteins hnRNP K and NS1-BP (Fig. 1a)^11,12^. Depletion of either hnRNP K and NS1-BP reduced the ratio of M2 to M1 mRNA and inhibited viral replication. Moreover, we further demonstrated that both hnRNP K and NS1-BP regulate M RNA splicing via a nuclear speckle-dependent mechanism. We found that NS1-BP is required for recruitment of M1 RNA to speckles along with promoting splicing and export of M mRNAs, while knockdown of hnRNP K causes a build-up of unspliced M1 RNA in these subnuclear structures^12^. This leads to a model in which M1 RNA is recognized by NS1-BP, which facilitates M1 RNA localization to nuclear speckles where hnRNP K then promotes M2 splicing (Fig. 1a).

NS1-BP was initially characterized as a nuclear speckle-associated protein that interacts with IAV protein NS1 during infection^13^. NS1-BP is a member of the kelch repeat superfamily of proteins, with an N-terminal BTB/POZ domain and 6 kelch repeats at the C-terminal predicted to fold into a β-propeller^14^. Like many kelch repeat proteins, NS1-BP is functionally diverse, being implicated in actin stabilization^15^, transcriptional regulation^16^, and signal transduction^17^; however, to date, the only connection of NS1-BP with splicing regulation has been with regards to the IAV M segment^11,12^. By contrast, hnRNP K contains 3 KH-type RNA-binding domains and has been previously shown to regulate numerous alternative splicing events in both host and viral contexts^18–21^. HnRNP K also localizes to nuclear speckles^22^ and interacts with NS1-BP in an RNA-independent manner, although RNA enhances the interaction^11^. Importantly, while our previous work set precedent for hnRNP K and NS1-BP co-regulating M2 splicing, it remains unclear how either protein mechanistically contributes to splicing regulation and whether these proteins co-regulate additional splicing events in a similar manner.

Here we use a combination of biochemistry, cell biology, and mutant viruses to identify new regulatory sequences controlling M1 to M2 splicing and define the mechanism of hnRNP K regulation of IAV M pre-mRNA splicing. Specifically, we find that NS1-BP and hnRNP K bind to the M transcript at adjacent sites just downstream of the M2 5′ splice site (5′ss). Association of hnRNP K with its cognate site leads to recruitment of NS1-BP and the U1 snRNP component of the spliceosome to the M2 5′ss. Importantly, mutation of the hnRNP K binding site reduces IAV M2 splicing and significantly attenuates viral replication. By contrast, NS1-BP associates weakly with a pyrimidine-rich element overlapping the 5′ss. This association appears to inhibit the use of the 5′ss in viral-infected cells, perhaps aided by the viral protein NS1. Thus, the balance of NS1, NS1-BP and hnRNP K binding to the M1 transcript controls the efficiency of M2 splicing. Strikingly, we also find that NS1-BP and hnRNP K coordinately regulate splicing of a set of host genes in the absence of NS1 and that some of the splicing events are susceptible to mis-regulation during viral infection. Therefore, our identification of the mechanism of hnRNP K/NS1-BP regulation of IAV M splicing provides potential new targets of antiviral intervention and also reveals novel activities of these proteins in mammalian cell biology.

## Results

### hnRNP K and NS1-BP regulate M1/M2 ratio in Influenza

In order to study the mechanism of hnRNP K and NS1-BP regulation of splicing, we first confirmed previous qPCR data, which showed that hnRNP K and NS1-BP regulate IAV M segment splicing^11^, using the orthogonal approach of primer extension. We infected A549 cells with the IAV strain A/PuertoRico/8/34 (PR8) for 12 h in the context of hnRNP K or NS1-BP siRNA knockdown (Fig. 1b) and assayed M segment splicing using an extension primer at position 903 (Fig. 1c-e). Because we are interested in mRNA changes exclusively, and the M1 primer extension product includes IAV mRNA and cRNA (the copy of the genomic negative strand used for replication), we also used an upstream 106 primer that can resolve the 10–13 nucleotide 5′ cap included on the mRNA (Fig. 1c, top). From this, we calculated the percentage of mRNA vs. cRNA in each condition (Fig. 1d), allowing us to determine how much of the M1 band resolved with the 903 primer was specifically mRNA. Comparing the M1 mRNA with the other M segment isoforms, we see that M1 to M2 mRNA ratio in mock cells is roughly equal (Fig. 1d, e, mock). By contrast, knockdown of either hnRNP K or NS1-BP results in a strong bias of M1 mRNA over M2, while M3 does not change significantly between conditions (Fig. 1c–e). Importantly, the changes we observe in PR8 M1 and M2 splicing as a result of hnRNP K and NS1-BP depletion are fully consistent with our previous observations made in A/WSN/33^6^ strain, confirming the generality of this regulatory program among influenza A viruses.

### hnRNP K and NS1-BP bind adjacent sites within the M1 intron

**Fig. 2:**
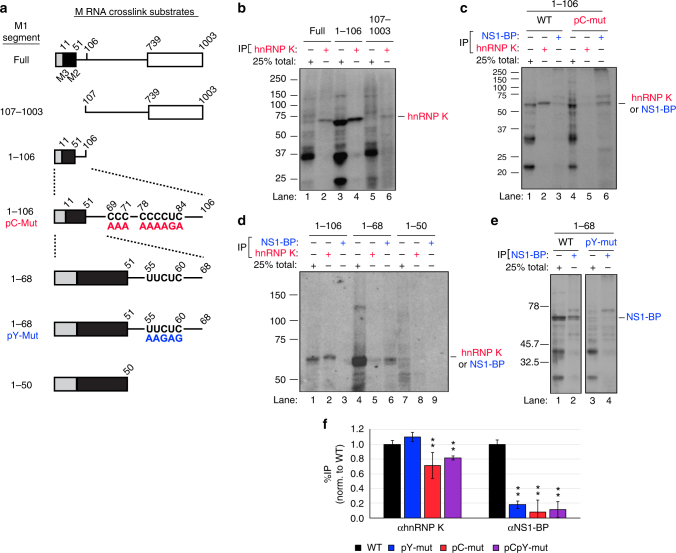
hnRNP K and NS1-BP bind adjacent sequences downstream of M2 5′ss. **a** Diagrams of M segment-derived RNA substrates used for UV-crosslinking assays. Names of each probe are indicated on the left. Boxes denote exons and the lines denote introns. Mutations of WT nucleotides are in color with hnRNP K associated mutations in red and NS1-BP associated mutations in blue. **b** UV-crosslinking of probes: Full, 1–106, and 107–1003. IP for hnRNP K. For total lanes 25% of the reaction was loaded. For IP lanes, indicated proteins were immunoprecipitated from RNase-treated cross-link reactions via primary antibody and 100% of bead eluate was loaded. Sizes of immunoprecipitated cross-link species are indicated on the right of the gel image. **c** UV-crosslinking of probes: 1–106 and 1–106 pC-mut. IP for hnRNP K and NS1-BP. **d** UV-crosslinking of probes: 1–106, 1–68, and 1–50. IP for hnRNP K and NS1-BP. **e** UV-crosslinking of probes: 1–68 and 1–68 pY-mut. IP for NS1-BP. **f** CLIP-PCR of full-length M segment probes cross-linked in nuclear extract and immunoprecipitated. RNA bound to immunoprecipitated protein was quantified using low-cycle, radiolabeled RT-PCR. Percent immunoprecipitation was calculated relative to total input RNA and values shown are normalized to percent immunoprecipitation of WT probe (Gel of RT-PCR products shown in Supplementary Fig. 2). Uncropped gels shown in Supplementary Fig. 5. Values are means ± s.d. from three independent experiments. For all experiments in manuscript, a minimum of 3 experiments were performed to insure appropriateness of statistical tests. Statistical significance was determined via two-tailed student’s *t* test, where *p < 0.1 and **p < 0.05 when comparing mock vs siRNA samples

As a first step to determining how hnRNP K and NS1-BP target M RNA for splicing, we characterized the interactions of hnRNP K and NS1-BP with M1 RNA. Previous data showed hnRNP K binding to M1 RNA via UV-crosslinking assays, but the binding sequence was not determined^11^. To identify the site(s) of hnRNP K binding to the M1 transcript, we made a series of truncations of the M1 mRNA (Fig. 2a, additional truncations in Supplementary Fig. 1) and carried out UV-crosslinking assays in which uniformly radiolabeled, in vitro transcribed RNA was incubated with nuclear extracts under splicing conditions (Fig. 2b–e). Identities of cross-linked species were confirmed via immunoprecipitation (IP). As shown in Fig. 2b, multiple proteins bind to the full-length M1 RNA (Full), including the ~65–70 kD hnRNP K (lane 2). While hnRNP K is not the most prominent protein bound to the full-length transcript, it binds robustly to the first 106 nucleotides (1–106; note in all these experiments migration of proteins following IP are slightly retarded compared to total lane as confirmed by using recombinant protein (Supplementary Fig. 1f)). By contrast, we observe reduced binding of hnRNP K to a construct lacking the first 106 nucleotides (lanes 2 and 6), or to other sub-fragments of the region 107–1003 (Supplementary Fig. 1b**)**. Previous studies have shown that hnRNP K binds preferentially to poly-cytosine (pC) tracts^23^. Notably, mutation of two pC tracts at nt 69–71 and 78–84, respectively (pC-mut, Fig. 2a) abrogates hnRNP K cross-linking to the 1–106 fragment (Fig. 2c, lanes 2 vs. 5). A similar result was also observed upon removal of the pC tracts by truncation at nucleotide 68 (Fig. 2a, d, lanes 2 vs. 5). Therefore, while hnRNP K may bind at some level to multiple sites along M1, we conclude that the pC tract within nucleotides 69–84 represent a major binding site for hnRNP K within M1. As this site is proximal to the regulated M2 5′ss, we focused on this binding site in subsequent studies below.

Interestingly, upon either mutation or deletion of the pC tracts, we observe a crosslinking signal for NS1-BP (Fig. 2c, d, lanes 3 vs. 6). This is surprising because NS1-BP is not known to contain any canonical RNA-binding domains^14^. Thus, our crosslinking result suggests NS1-BP either binds RNA through a non-canonical interaction, a phenomena supported by recent studies^24,25^, or is in extremely close proximity to the RNA via protein-protein interaction with another RBP(s). Upon further truncations of the M1 transcript, we determined the NS1-BP binding to be enriched within the M1 intron between nucleotides 51 and 68 (Fig. 2a, d, lanes 4, 6, and 9, Supplementary Fig. 1). Mutational analysis of nucleotides 50-68 revealed NS1-BP cross-linking to be dependent on a poly-pyrimidine (pY) tract at nt 55–60 (Fig. 2e, lanes 3–4; additional mutations in Supplementary Fig. 1). To confirm that the NS1-BP cross-link species is not the result of antibody cross-reaction, we looked to see if other RNPs identified to interact with NS1-BP and/or bind to poly-pyrimidine sequences produce the same crosslinking species (Supplementary Fig. 1e). In all cases, the specific cross-link species is unique to NS1-BP antibody.

Given the potential caveats with using highly truncated and radiolabeled RNAs in UV crosslinking, we also used a cross-link immunoprecipitation RT-PCR (CLIP-PCR) approach with full-length M1 RNA to further assessed the importance of the pC and pY tracts in the recruitment of hnRNP K and NS1-BP. Following crosslinking, hnRNP K and NS1-BP were immunoprecipitated and bound M1 probes were quantified using RT-PCR (Fig. 2f, gel shown in Supplemental Fig. 2). Consistent with the crosslinking results of the RNA fragments, mutation of the pY markedly reduces precipitation of M1 with the NS1-BP antibody, while having no effect on M1 association with hnRNP K. By contrast, mutation of the pC tract reduces precipitation of M1 with hnRNP K, supporting the conclusion that the pC tract is the primary binding site for hnRNP K within the M1 transcript. Interestingly, however, we do find that NS1-BP association with M1 is also reduced by the pC mutation (Fig. 2f). Given the known interaction between NS1-BP and hnRNP K, we interpret this data as revealing that hnRNP K bound to the pC sequence helps stabilize the binding of NS1-BP to adjacent pY tract. The lack of apparent NS1-BP signal in the crosslinking to the radiolabeled 1–106 (Fig. 2c, d) is likely due to the overwhelming labeling of the pC tract with 32P-CTP and signal from the tight interaction with hnRNP K.

**Fig. 3:**
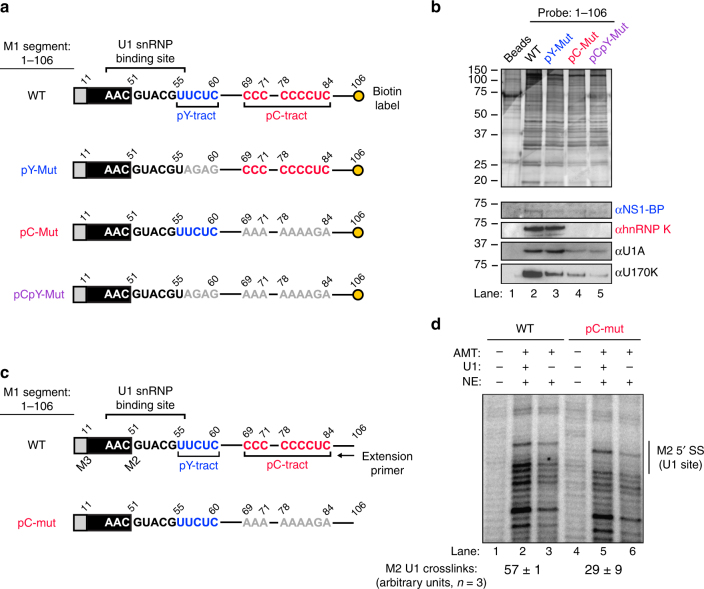
hnRNP K binding site promotes U1 snRNP binding. **a** Diagram of in vitro transcribed, biotinylated RNA affinity baits derived from IAV M segment mRNA. Blue text corresponds to NS1-BP binding, red text corresponds to hnRNP K binding, based on data from Fig. 2. Grey text corresponds to mutated nucleotides. **b** RNA affinity purification of proteins bound to baits: 1–106 WT, 1–106 pY-mut, 1–106 pC-mut, 1–106 pCpY-mut. RNA probes were incubated in JSL1 nuclear extract and isolated with streptavidin beads. Bead eluates were resolved via SDS-PAGE and either silver stained (top panel) or immunoblotted for specific proteins (bottom panels). Molecular weights are indicated to left of each blot. **c** Diagram of in vitro transcribed AMT psoralen cross-linking substrates derived from IAV M segment mRNA. Position of extension primer is shown below. **d** Radiolabeled primer extension of M segment RNA substrates after AMT psoralen cross-linking. RNA substrates were incubated with JSL1 nuclear extract ± presence of U1 snRNP, supplemented with AMT psoralen, and irradiated with 365 nm UV light. Cross-link products were visualized by primer extension. Identity of cross-link products was determined using dideoxy sequencing (Supplementary Fig. 3). M2 U1 crosslinking quantification is in arbitrary units representing the signal of bands at the M2 5´ss in the presence of U1 ( + U1) divided by the sum of bands in the presence and absence of U1. Values are means ± s.d. from three independent experiments. Uncropped gels shown in Supplementary Fig. 5

As an orthogonal method to further assess association of hnRNP K and NS1-BP with the M1 RNA, we biotinylated fragments of M1, incubated with nuclear extract and assessed co-associated proteins by western blot (Fig. 3a, b). Although this assay detects both direct as well as indirect association with RNA, the results are entirely consistent with the CLIP-PCR results. Specifically, NS1-BP association with the 1–106 fragment is reduced by both the pY or pC mutations, alone or in combination, while hnRNP K binding is only reduced upon mutation of the pC tract. Since we ultimately sought to understand how hnRNP K and NS1-BP influence removal of the M1 intron, we also blotted the affinity purification samples for core components of the spliceosome. Specifically, we assessed the presence of large ribonucleoprotein, U1 snRNP, which binds the 5′ss in the first steps of intron removal^9^. The M2 5′ss is the one splicing signal completely contained within the constructs used in the RNA-affinity experiments. Strikingly, we find clear association of U1 snRNP-specific proteins U1A and U170 K with the M1 1–106 construct in a manner that directly mirrors the association of hnRNP K (Fig. 3b). The dependence of U1A and U1 70K association on the pC tract, which is well outside the 5′-ss recognition region of the U1 snRNP, implies that the presence of hnRNP K promotes binding of the U1 snRNP to the M2 5′-ss.

To test U1 snRNP association more directly, we turned to AMT psoralen cross-linking, which captures RNA–RNA basepairs formed between the U1 snRNP RNA component, U1 snRNA, and the 5′ss. As shown in Fig. 3c, d, AMT psoralen induced cross-links of WT M1 RNA as visualized by termination of primer extension (Fig. 3c, lanes 2–3). Location of the M2 5′ss region was determined by parallel dideoxy-sequencing ladders (Supplementary Fig. 3a). Comparison of lanes 2 and 3 reveals a reduction in signal at the M2 5′ss upon depletion of U1 snRNA to ~30% of wild-type levels (Supplementary Fig. 3b). Subtraction of the cross-link signal in lane 3 from lane 2 yields a value for U1-dependent cross-links at the M2 5′ss (Fig. 3d, full explanation of quantification is addressed in methods). Consistent with the RNA affinity results, the same analysis of U1 cross-linking at the M2 5′ss in the context of the pC-mut shows that U1 crosslinking is decreased by 51% (Fig. 3c, d, lanes 2 vs. 5). Taken together, we conclude that the pY tract adjacent to the M2 5′ss is required for NS1-BP association with the M1 intron in conjunction with hnRNP K, while the presence of the pC tract results in direct binding of hnRNP K and recruitment of the U1 snRNP to the M2 5′ss.

### hnRNP K and NS1-BP-binding sites regulate M2 splicing

**Fig. 4:**
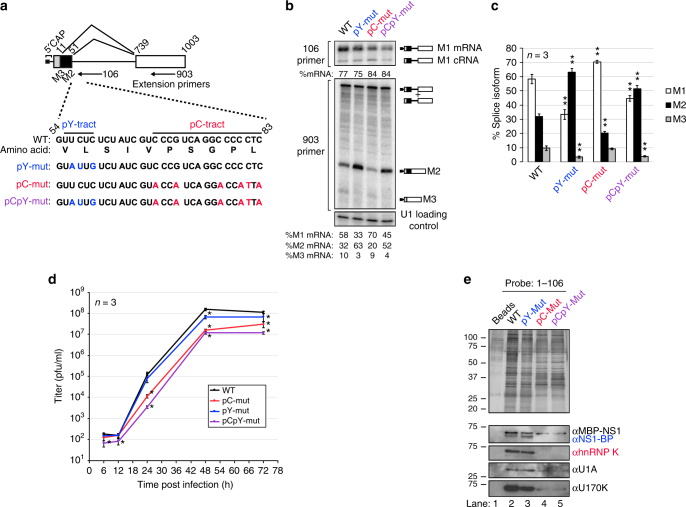
hnRNP K and NS1-BP-binding sites differentially regulate IAV PR8 M segment splicing and replication. **a** Diagram of IAV PR8 M segment mRNA with possible splice isoforms and extension primer positions labeled. Labels are as in Fig. 1. **b** Primer extension of PR8 M segment antisense RNA after infection in A549 cells at moi = 2. Extension primers used are indicated on the left and band identities on the right of the gel image. **c** Quantification of mRNA primer extension products represented as percentage signal of each isoform relative to signal of total isoforms (M1 + M2 + M3). Values are means ± s.d. from three independent experiments. Statistical significance was determined via two-tailed student’s *t* test, where *p < 0.1 and **p < 0.05 when comparing mock vs siRNA samples. **d** A549 cells were infected with WT and mutant PR8 at moi = 0.01 and assayed for plague forming units over 72 h. Values are means ± s.d. from three independent experiments. *p < 0.05. **e** RNA affinity purification of proteins bound to baits: 1–106 WT, 1–106 pY-mut, 1–106 pC-mut, 1–106 pCpY-mut (diagram of probes in Fig. 3a). RNA probes were incubated in JSL1 nuclear extract supplemented with recombinant, MBP-tagged NS1 and isolated with streptavidin beads. Bead eluates were resolved via SDS-PAGE and either silver stained (top panel) or immunoblotted for specific proteins (bottom panels). Molecular weights are indicated to left of each blot. Uncropped gels shown in Supplementary Fig. 5. Statistical significance was determined via two-tailed student’s *t* test, where *p < 0.1 and **p < 0.05 when comparing mock vs siRNA samples

Having identified binding sites for hnRNP K and NS1-BP, we next sought to determine the functional relevance of the pY and pC sequences to M1 to M2 splicing using a previously established reverse genetics system to engineer mutations in the pY and/or pC tracts in the PR8 IAV strain (Fig. 4a)^26^. Of note, these mutations differ from the ones used in the binding studies so as to preserve the amino acid sequence of the M segment; however, we confirmed these mutations still impact hnRNP K and NS1-BP binding (binding data in Supplementary Fig. 4). We measured M segment splicing of WT and PR8 mutants at 6 h post infection in A549 cells using primer extension as in Fig. 1 (Fig. 4a–c). Consistent with the demonstration that hnRNP K binding to the pC tract promotes recruitment of the U1 snRNP, mutation of the pC-tract results in a 13% decrease in the production of spliced M2 and a corresponding increase in the unspliced M1 (Fig. 4b, lane 3). Similar results were also observed 12 h post-infection (Supplementary Fig. 4c). Importantly, the pC mutant virus also exhibits a significant defect in replication (*p* < 0.05), underscoring the importance of appropriate splicing for the viral cycle (Fig. 4d).

**Fig. 5:**
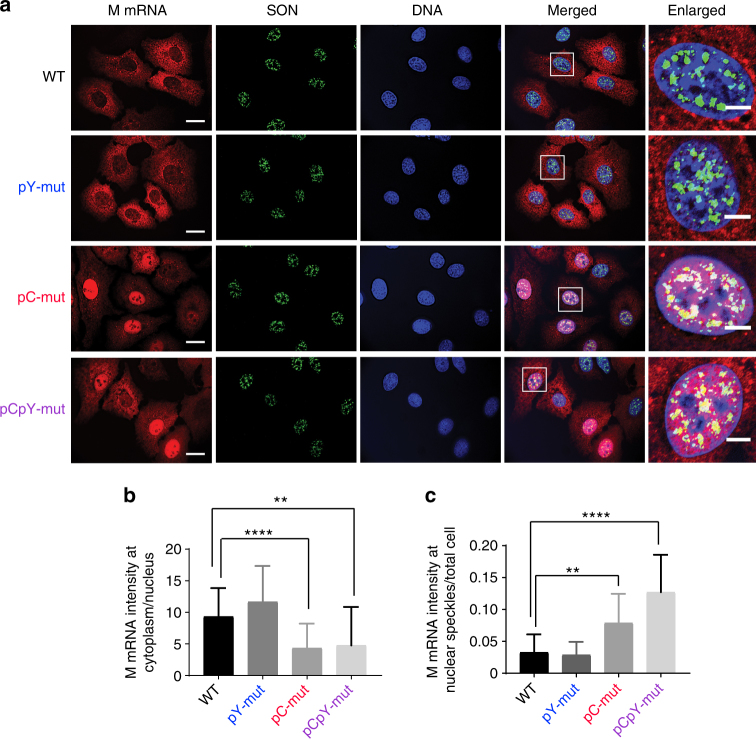
Mutation of hnRNP K but not NS1-BP-binding sites alters M mRNA localization. **a** A549 cells were infected with wild type (WT) and mutant A/WNS/33 viruses at MOI 5. After 8.45 h infection, cells were subjected to RNA-FISH combined with immunofluorescence to detect M mRNA and SON protein. SON protein is a marker for nuclear speckles. Scale bar, 20 µm. The marked rectangular region is enlarged and shown in the right-most panel. Scale bar, 5 µm. Images are representative of at least 12 images from biological triplicates. **b** Quantification of M mRNA intensity in the cytoplasm (C) and in the nucleus (N) expressed as C/N ratios. Values are mean ± s.d. measured in at least 25 cells. **c** Quantification of M mRNA intensity at nuclear speckles with respect to total cell intensity. Values are mean ± s.d. measured in at least 25 cells. Statistical significance was determined via two-tailed student’s *t* test, where **p < 0.01 and ****p < 0.001 when comparing mock vs. mutant samples

Surprisingly, however, we find that mutation of the pY sequence enhances splicing at the M2 5′ss, as shown by a sharp increase in M2 production relative to M1 either on its own or in combination with the pC mutation (Fig. 4b, lane 2 and 4; and Fig. 4c). While this is opposite to the effect of NS1-BP depletion (Fig. 1d, e), we note that the pY tract overlaps with the U1 and U6 recognition sites at the 5′ss. Therefore, we conclude that association of NS1-BP with the pY sequence likely sterically hinders formation of the spliceosome on the M2 5′ss. However, it was still unclear why the pY mutation does not phenocopy the depletion of NS1-BP. Notably, in Fig. 3, we did not detect NS1-BP bound to the pY mutant RNA, but this was in the absence of the viral NS1 protein that is present in these infection experiments. To ask if NS1 alters association of NS1-BP with the M1 RNA, we repeated the RNA-affinity experiment in the presence of recombinant NS1. Strikingly, we find that in the presence of NS1, association of NS1-BP with the M1 transcript is no longer dependent on the pY sequence, although it is still dependent on the presence of hnRNP K (Fig. 4e). Consistently, by immunofluorescence, we find no difference in the trafficking of wild type versus pY mutant M1 to speckles or the cytoplasm (Fig. 5), which we have previously shown to be dependent on NS1-BP and NS1^12^. Thus, we conclude that NS1 stabilizes NS1-BP association with M1 even in the absence of the pY sequence. On the other hand, both the pC and pCpY mutant M1 exhibit the same retention in the speckles and depletion from the cytoplasm as we have observed previously upon depletion of hnRNP K^12^. Importantly, the increased speckle localization of the pC and pCpY mutant is not merely a consequence of increased nucleoplasmic concentrations, as other perturbations that retain M mRNA in the nucleus, such as NS1 knockout, do not increase nuclear speckle localization^12^. Thus, these data further indicate that hnRNP K functions through the pC tract. Moreover, the loss of cytoplasmic trafficking provides an explanation for the inhibited replication of the pC and pCpY mutant viruses (Fig. 4d). Taken together, these data provide explanation for how hnRNP K and NS1-BP binding to specific M segment RNA elements directly results in regulation of IAV mRNA splicing, mRNA localization, and replication.

### NS1-BP and hnRNP K co-operatively regulate host splicing

**Fig. 6:**
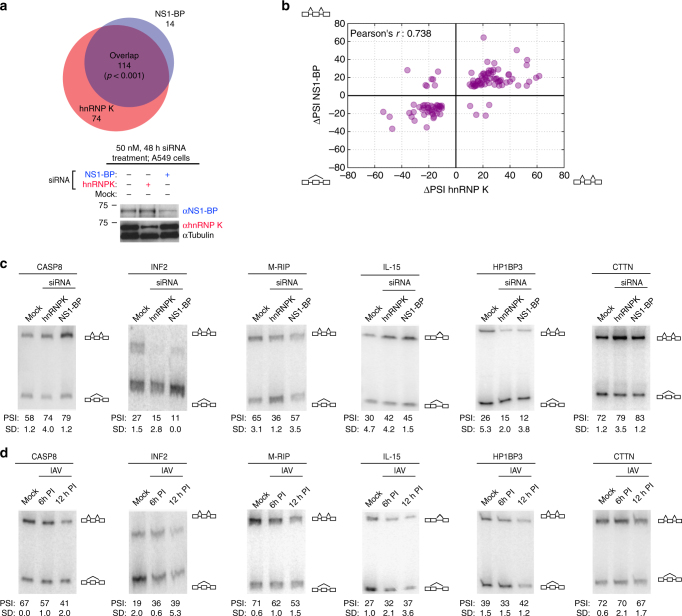
hnRNP K and NS1-BP co-operatively regulate host alternative splicing events. A549 cells were mock transfected or treated with 50 nM siRNA targeted for hnRNP K or NS1-BP in triplicate. Total cell RNA was then extracted and subjected to RASL-seq. **a** Overlap of hnRNP K and NS1-BP responsive alternative splicing events with ∆PSI ≥ |10| and *p* < 0.05 (students *t* test). Overlap significance determined by Fisher’s exact test. Whole cell extracts were immunoblotted for hnRNP K and NS1-BP (and Tubulin loading control) to determine siRNA knockdown efficiency. Molecular weights are indicated to left of each blot. **b** Correlation plot of ∆PSI values of alternative splicing events regulated by hnRNP K and NS1-BP (overlap in panel **a**). Positive values indicate alternative exon inclusion and negative values indicate alternative exon skipping upon knockdown of hnRNP K or NS1-BP. **c** Representative validations of hnRNP K and NS1-BP-regulated alternative splicing events using low-cycle, radiolabeled RT-PCR visualized via PAGE. Quantification represent signal of upper inclusion product divided by total signal of upper inclusion product and lower exclusion product (complete list of validations in Supplementary Table 1). **d** Low-cycle, radiolabeled RT-PCR analysis of validated hnRNP K and NS1-BP regulated splicing events during IAV infection at moi = 2. PSI and SD represent 3 independent experiments. Uncropped gels shown in Supplementary Fig. 5

Although NS1-BP has been studied with respect to IAV and the viral NS1 protein, its role in uninfected cells has not been well characterized. Our observation that NS1-BP associates with RNA in the absence of virus caused us to ask if NS1-BP might have activity as a regulator of splicing of human genes. We therefore carried out a quantitative analysis of splicing of ~5500 known alternative exons in A549 cells in the absence or presence of knockdown of NS1-BP or hnRNP K using the previously described RASL platform^27^. Briefly, cells were depleted of NS1-BP or hnRNP K as in Fig. 1 and then total RNA was collected and subjected to RASL-Seq (see Methods section) to score for inclusion (Percent Spliced In or PSI) of interrogated exons. Exons that exhibited a statistically significant (two-tailed *t* test, *p* < 0.05) change of >10 PSI between WT and knockdown cells (|ΔPSI| > 10) were considered to be spliced in a protein-dependent manner. Consistent with the previously described role of hnRNP K as a splicing regulator, we observed that splicing of ~200 out of the ~5500 exons surveyed are dependent on hnRNP K (Fig. 6a, Supplementary Data 1). We also found ~120 exons that are regulated in an NS1-BP-dependent manner (Fig. 6a, Supplementary Data 1). Remarkably, almost all of the NS1-BP-dependent exons were also dependent on hnRNP K (Fig. 6a), and were regulated in the same direction by both hnRNP K and NS1-BP (Fig. 6b). Indeed, RT-PCR failed to confirm the few instances in which the RASL-Seq analysis suggested that NS1-BP regulated an exon apart from hnRNP K (Fig. 6a, 14 NS1-BP only exons) or in an opposite direction (Fig. 6b, upper left or bottom right quadrant, Supplementary Table 1). By contrast, instances of predicted co-regulation by hnRNP K and NS1-BP were well validated by RT-PCR (Fig. 6c, Supplementary Table 1). The limited scope of splicing events interrogated by RASL-seq makes it unfeasible to assess sequence enrichment within the hnRNP K and NS1-BP co-regulated genes. However, we do see obvious proximal pY and pC-tracts downstream of hnRNP K and NS1-BP-binding sites downstream of several regulated 5′ss in host genes (see Discussion section). Taken together, these data reveals a previously unappreciated function for NS1-BP in host gene regulation and suggests that IAV has hijacked a pre-existing widespread cellular splicing regulatory relationship between hnRNP K and NS1-BP to carry out its own M1 to M2 splicing.

Having established that IAV protein, NS1, can contribute to hnRNP K and NS1-BP RNA association (Fig. 4e), we wondered if IAV infection has any influence on hnRNP K and NS1-BP dependent splicing events. Remarkably, we find that IAV infection induces misregulation of hnRNP K and NS1-BP regulated splicing events. Specifically, we assessed 6 validated hnRNP K and NS1-BP co-regulated splicing events in the context of IAV infection via RT-PCR (Fig. 6d) and observed alterations in 4 out of 6. Notably, the impact of IAV infection on splicing implies two distinct mechanisms. For CASP8 and INF2 the impact of IAV was opposite of what we observed upon hnRNP K and NS1-BP knockdown, while splicing of M-RIP and IL-15 in IAV-infected cells phenocopied hnRNP K or NS1-BP depletion. Together, these data show that IAV influences, with some specificity and variety, hnRNP K and NS1-BP regulated splicing events, suggesting new paradigms for the impact of IAV infection upon host gene regulation (see Discussion section).

## Discussion

**Fig. 7:**
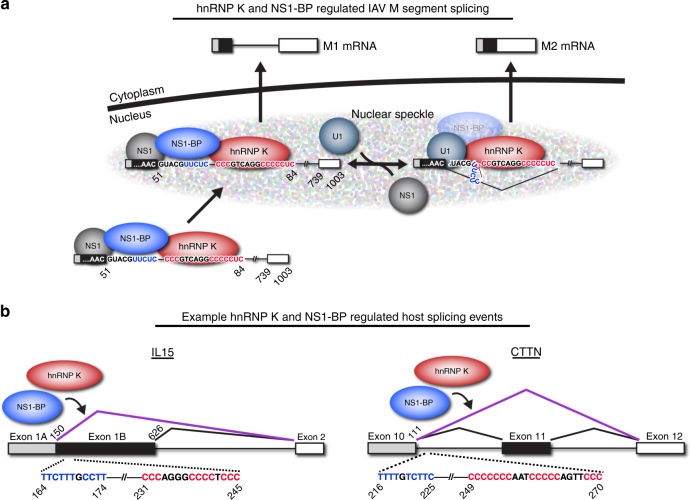
Viral and host models for hnRNP K- and NS1-BP-regulated splicing. **a** Model of hnRNP K and NS1-BP splicing regulation of influenza M segment splicing (as described in text). **b** Putative model of hnRNP K and NS1-BP regulated splicing of host RNA

Combining our previous knowledge of M segment splicing at the level of RNA localization^12^ and our current data that explores hnRNP K and NS1-BP interaction with M RNA, we propose a detailed model of M segment splicing during IAV infection (Fig. 7a). First, outside the speckle we suggest that NS1, NS1-BP and hnRNP K bind to the M1 transcript in a manner that stabilizes NS1-BP association with pY-tract directly downstream of the M2 5′ss (Fig. 7a, bottom-left complex**)**. This complex likely prevents inappropriate splicing of the transcript until such time that the transcript is localized to speckles. Once in the speckle (Fig. 7a, upper-left complex), we propose that the high concentration of U1 snRNP and/or dissociation of NS1 results in a remodeling in which hnRNP K recruits U1 snRNP to the M2 5′ss to promote splicing (Fig. 7a, upper-right complex). In this model, weakening of the NS1-BP interaction over the 5′ss by mutation of the pY tract is not sufficient to destabilize the initial NS1/NS1-BP/hnRNP K complex (Fig. 7a, bottom-left complex) but is predicted to shift the equilibrium inside the speckle in the forward direction (Fig. 7a, upper-left to upper-right complex), whereas mutation of the pC tract or depletion of hnRNP K would have the opposite effect by failing to promote U1 snRNA binding, thereby limiting splicing (Fig. 7a).

This model takes into consideration previous findings showing that upon IAV infection NS1-BP is re-distributed throughout the nucleus before returning to nuclear speckles^13^, that depletion of either NS1-BP or NS1 results in reduced recruitment of M transcript to speckles^12^, and that NS1 is a direct RNA binder^28^ and splicing repressor^29,30^. In addition, this model incorporates all of our binding data, including the dependence of hnRNP K binding on the pC tract with or without NS1 (Figs. 2, 3b and 4e), and the fact that NS1 and hnRNP K can together stabilize indirect association of NS1-BP with M1 even in the absence of the pY tract (Fig. 4e). Finally, the functional impact of the pY and pC mutations (Fig. 3c, d, Fig. 4a-d, Fig. 5), also are consistent with this model in that the pY mutation increases splicing once in speckles, whereas mutation of pC decreases production of M2.

Importantly, the model we propose implies a dual function of NS1-BP, in which NS1-BP not only regulates mRNA localization as previously shown, but also directly contributes to splicing regulation. Such a two-function model is consistent with the fact that mutation of the pY-tract disrupts direct association of NS1-BP with M1 (Figs. 2e, 3b, Supplementary Fig. 4) and mis-regulates splicing (Fig. 4), but does not alter the transport activity of NS1-BP (Fig. 5). Moreover, this model accounts for why mutation of the pY-tract has a different impact on M1/M2 production as does depletion of NS1-BP (Fig. 4e). In sum, our data, together with previous studies, support a role for NS1-BP in controlling the spatial and temporal access of the M transcript to the splicing machinery through both subnuclear localization as well as direct competition.

It is possible that the displacement of NS1-BP from the pY is itself sufficient to render the M2 5′ss accessible to the U1 snRNP, however, the fact that we observe increased production of M2 in the pY mutant versus the pCpY double mutant (Fig. 4) suggests that hnRNP K plays an active role in recruiting U1 snRNP. Importantly, hnRNP K has previously been shown to interact with components of the U1 snRNP, providing a possible mechanism for hnRNP K-dependent U1 snRNP recruitment^31,32^. Finally, we do not rule out the possibility that NS1-BP might also help to promote U1 snRNP recruitment (Fig. 7a, upper-right complex). While NS1-BP does not associate strongly with M1 RNA in the absence of the pY tract and NS1, recent work from our groups have shown that NS1-BP does interact with the U1A component of the U1 snRNP in an RNA-dependent manner (Zhang and Fontoura, personal communication), suggesting that this interaction could contribute to the overall stability (Fig. 7a, upper-right complex).

In comparison to other examples of alternative splicing, hnRNP K binding within the M1 intron resembles previous work showing that hnRNP K binds within the intron of chicken β-tropomyosin pre-mRNA to promote exon inclusion^21^. Moreover, specific subsets of intron-retained transcripts have been proposed to be regulated post-transcriptionally, comparable to the M segment example^33–35^. Indeed, we identified over 100 splicing events that are co-regulated by hnRNP K and NS1-BP (Fig. 6). While hnRNP K has previously been shown to regulate splicing^18–21^, nor NS1-BP, or any member of the kelch repeat superfamily, have ever been described as a broad splicing regulator. Strikingly, NS1-BP regulated splicing events almost exclusively overlapped hnRNP K-regulated events, indicating widespread cooperation between these two proteins. Within these co-regulated events, we see potential hnRNP K- and NS1-BP-binding motifs downstream of regulated 5′ss (Fig. 7b). In particular, in host genes Interleukin 15 (IL15) and cortactin (CTTN), we find pY- and pC-tracts adjacent to one another and downstream of the promoted 5′ss; a configuration that is exactly reminiscent of the M segment (compare Fig. 7a, b). Importantly, while the alternative 5′ss splicing example of IL15 closely resembles the M segment, the CTTN example represents a cassette exon, suggesting that hnRNP K and NS1-BP could regulate a broad set of diverse splicing events using similar sequence motifs.

Although due to the relatively small sample size, gene ontology analysis did not suggest any significantly enriched classes of genes represented in the hnRNP K and NS1-BP co-regulated exon, several of the pre-mRNAs co-regulated by NS1-BP and hnRNP K encode proteins known to have a role in apoptosis and immunity (CASP8 and IL15). Since these are pathways regulated during virus infection, it is possible that this co-regulatory splicing of host pre-mRNAs by NS1-BP and hnRNP K may impact immunity and cell death, which is a topic for future investigation. Interestingly, we find that IAV infection resulted in the mis-splicing of several of the hnRNP K- and NS1-BP-regulated transcripts we tested. In some cases IAV infection mimicked depletion of hnRNP K and NS1-BP, while in other cases the activities of these proteins seemed to be enhanced (Fig. 6d). Further investigation will be required to fully understand the set of mechanism(s) by which viral infection alters hnRNP K/NS1-BP-dependent splicing; however, these data suggest that alternative splicing may be a critical component of a host-viral response to IAV. Importantly, such a conclusion is supported by recent work by others demonstrating reovirus influence on host splicing^36^, HCMV regulation of alternative poly-adenylation^37^, and HSV regulation of splicing and poly-adenylation^38^.

## Methods

### Cell culture

Human lung adenocarcinoma epithelial cells (A549) were cultured in RPMI 1640 (Corning: 10-040-CV), 10% heat-inactivated FBS (Gibco: 16000-044), and 100 units per ml Pen/Strep antibiotics. MDCK cells were cultured in high-glucose DMEM (Corning: 10-013-CV), 10% heat-inactivated FBS (Gibco) and 100 units per ml Pen/Strep antibiotics. All cells were maintained at 37 °C with  5% CO_2_. Cells were tested negative for mycoplasma. Cell lines were obtained and authenticated by ATCC (A549: CCL-185, MDCK: PTA-6500).

### Viruses

Wild type and mutant M sequences from either A/Puerto Rico/8/1934 (PR8) or A/WSN/1933 (WSN) strains were gene synthesized in vitro (ThermoFisher Scientific) and cloned into PDZ vector SapI restriction site, respectively. Standard reverse genetics were used to rescue each individual virus as previously described^26^, followed by plaque purification and propagation in MDCK cells. Finally, viral titers were determined through plaque assay calculated as the average of replicates and full genome deep sequencing was conducted on the Illumina MiSeq platform to confirm viral sequences.

### RNA interference and transfections

siRNA was purchased from Dharmacon (NS1-BP: SMARTpool, M-016604-02, hnRNP K: SMARTpool, M-011692-00). 2 × 10^5^ A549 cells (6-well format) were transfected with 50 nM siRNA using 1.5 µl RNAiMAX (ThermoFisher: 56532) per 25 pmol siRNA and incubated for 48 h in antibiotic-free media. Knockdown efficiency was assessed via Western blot.

### Infections

For infections, 8 × 10^5^ A549 cells (6-well format) were washed with PBS and inoculated with 200 µl virus diluted in PBS•BA (DPBS with Ca and Mg (Corning: 21-031-CV), 0.2% BSA (Lampire: 7500810), and 100 units per ml Pen/Strep antibiotics) for 1 h at room temperature (22 °C). Cells were then washed with PBS and incubated in Infection media (1 × MEM, 0.2% BSA, 10 mM HEPES buffer, 0.12% NaHCO_3_, 100 units per ml Pen/Strep antibiotics, and 0.2 µg/ml TPCK trypsin (Sigma: SLB58956)) at 37 °C with 5% CO_2_ until desired time-point. At cell collection, wells were rinsed with PBS and cells were either pelleted and lysed with RIPA buffer for Western blot analysis or RNA was extracted using RNA-Bee (amsbio: CS-501B).

### Primer extension

A concentration of 1.5 µg input RNA was hybridized with excess ^32^P-labeled reverse primer (sequences in Supplementary Data 2) in H_2_O by boiling 5 min, cooling to 4 °C for 4 min, and equilibrating at 45 °C for 4 min. Pre-warmed 2 × Ext-Buffer (100 mM Tris-HCl pH 8.0, 80 mM KCl, 12 mM MgCl_2_, 20 mM DTT, 1 mM dNTPs, and 50 U MMLV-RT (Thermofisher: 28025013)) was added to each reaction and incubated at 45 °C for 90 min. Reactions were denatured by boiling in formamide loading buffer and resolved on 5% denaturing poly-acrylamide (acrylamide/bis 19:1, BioRad: 1610144) gels.

### Antibodies

Antibody concentrations for immunoprecipitations and immunoblotting were determined empirically. All immunoblot antibodies were diluted as specified in 5% (w/v) BSA-TBST. Antibodies were purchased and used as follows: NS1-BP (Bethyl A302-879A, 1:2000 immunoblot, 4 µg IP), hnRNP K (Abcam ab39975, 1:1000 immunoblot, 4 µg IP), hnRNP E2 (Abcam ab77323, 2 µg IP), U2AF65 (Sigma U4758, 8 µg IP), hnRNP A1 (Abcam ab5832, 4 µg IP), PTBP1 (EMD MABE986, 1:1000 immunoblot, 4 µg IP), U1A (Abcam ab55751, 1:1000 immunoblot) U170K (Abcam ab51266, 1:1000 immunoblot), Tubulin (Abcam ab6046, 1:10,000 immunoblot), and NS1 (gift from García-Sastre lab, 1:1000 immunoblot).

### Ultraviolet crosslinking

UV-crosslinking assays were performed as previously described using JSL1 nuclear extracts^12^. M1 RNA substrates were generated from linearized plasmids and PCR fragments (sequences in Supplementary Data 2) using T7 polymerase and ^32^P-CTP to label the RNA throughout its length. Probes were specifically labeled with a labeled to unlabeled cytosine ratio of 1:30 in Fig. 1b. In all other cross-linking experiments hot to cold ratios were adjusted so that probes had equal ^32^P-CTP/mol probe. Immunoprecipitations after crosslinking were performed overnight in 400 µl RIPA buffer at 4 °C rotating end-over-end.

### CLIP-PCR

CLIP-PCR experiments were performed as follows. Unlabeled in vitro transcribed full-length M1 RNA probes were UV-cross-linked with JSL1 nuclear extract as described above (Methods section, Ultraviolet crosslinking). Post crosslinking 1% of input was removed before reaction was immunoprecipitated overnight in RIPA buffer. Immunoprecipitated complexes were purified using Invitrogen magnetic Dynabeads (Cat #10004D). RNA was then extracted using phenol chloroform isoamyl alcohol and EtOH precipitation. All samples were then subjected to low-cycle radiolabeled RT-PCR. A common PCR cycle number where all samples were within the linear detection range of the RT-PCR assay was determined empirically (Supplementary Fig. 2). Immunoprecipjtation percentage was calculated after dividing densitometry signal for immunoprecipitated samples by total input signal and multiplying by 100. Immunoprecipjtation percentage values were then normalized to values calculated for the WT probe.

### RNA affinity

RNA affinity experiments were performed as previously described^39^. Briefly, in vitro transcribed RNA probes generated from PCR templates (sequences in Supplementary Data 2) were biotin labeled using a kit (Pierce 20160) according to manufacturer’s instructions. 25 pmols of labeled RNA was incubated with 100 µg JSL1 nuclear extract under splicing conditions and bound to 30 µl streptavidin beads (Pierce: 53114). For NS1 experiments, 750 ng of recombinant MBP-tagged NS1 was added to nuclear extract before addition of RNA probe. RNA-protein-bead complexes were eluted in 2 × SDS loading buffer and resolved with 10% SDS-PAGE (acrylamide/bis 37.5:1, BioRad: 1610158). Eluate was visualized using Western blot and Biorad Silver Stain Plus Kit (cat #: 1610449).

### AMT psoralen crosslinking

Cross-linking reactions were performed as previously described with slight modifications^40^. Briefly, splicing complexes were assembled for 20 min at 30 °C in JSL1 nuclear extracts ± U1 snRNP. U1 snRNP was depleted by RNase H cleavage of U1 snRNA using a complimentary oligonucleotide (5′-TTCAGGTAAGTACTCA-3′) (Supplementary Fig. 3). AMT psoralen (Sigma, A4330) was added to each reaction to a final concentration of 40 µg/ml. Reactions were irradiated with 365 nm light for 10 min on ice. Cross-linked products were detected using ^32^P-labeled primer extension at 106 nt position (sequences in Supplementary Data 2). Products were resolved with 15% PAGE (acrylamide/bis 19:1, BioRad: 1610144) and nucleotide positions were determined using a dideoxy-sequencing ladder with the same primer (Supplementary Fig. 3). Arbitrary U1 cross-link units were determined by densitometry. Briefly, signal at putative U1 snRNA-binding sites when U1 snRNP was depleted was subtracted from corresponding signal when U1 snRNP was not depleted and then divided by total signal when U1 was present.

### RNA FISH and immunofluorescence

RNA FISH and immunofluorescence were performed as we have recently described^12^.

### RASL-seq and RT-PCR

RASL-seq was performed as previously described using a set of probes that interrogate ~5600 specific alternative splicing events^27,41,42^. Total RNA was harvested from A549 cells 48 h post 50 nM siRNA treatment in triplicate. RASL libraries were generated, barcoded, and sequenced on a HiSeq 2000. RASL events were then filtered for any event with an average read depth of 10 across all samples (Supplementary Data 1). Significant splicing events were considered any event with a *p*-value < 0.05 and ΔPSI value > |10|. Validations of splicing events were determined using ^32^P-labeled, low-cycle, RT-PCR with sequence-specific primers (sequences in Supplementary Data 2).

### Ultraviolet crosslinking of recombinant protein

UV-cross-linking assays were performed as previously described using JSL1 nuclear extracts^12^. M1 RNA substrates were generated from linearized plasmids and PCR fragments (sequences in Supplementary Data 2) using T7 polymerase and ^32^P-CTP to label the RNA throughout its length. Labeled transcripts were incubated with 500 ng recombinant protein under splicing conditions for 30 min at 30 °C. Immunoprecipitations after cross-linking were performed overnight in 400 µl RIPA buffer at 4 °C rotating end-over-end.

### Data availability

The authors declare that the data supporting the findings of this study are available within the article and its Supplementary Information files, or are available from the authors upon request.

## Electronic supplementary material

Supplementary Information

Description of Additional Supplementary Files

Supplementary Data 1

Supplementary Data 2
