# Supplementary material for: Co-regulatory activity of hnRNP K and NS1-BP in influenza and human mRNA splicing
Source: Nat Commun. 2018 Jun 19;9:2407. doi: 10.1038/s41467-018-04779-4 (PMC6008300; doi:10.1038/s41467-018-04779-4)
Supplement: Supplementary file 1 — Supplementary Information [file 41467_2018_4779_MOESM1_ESM.pdf]

Co-Regulatory Activity of hnRNP K and NS1-BP in Influenza and Human mRNA Splicing

Matthew G Thompson, et. al.

Supplementary Information

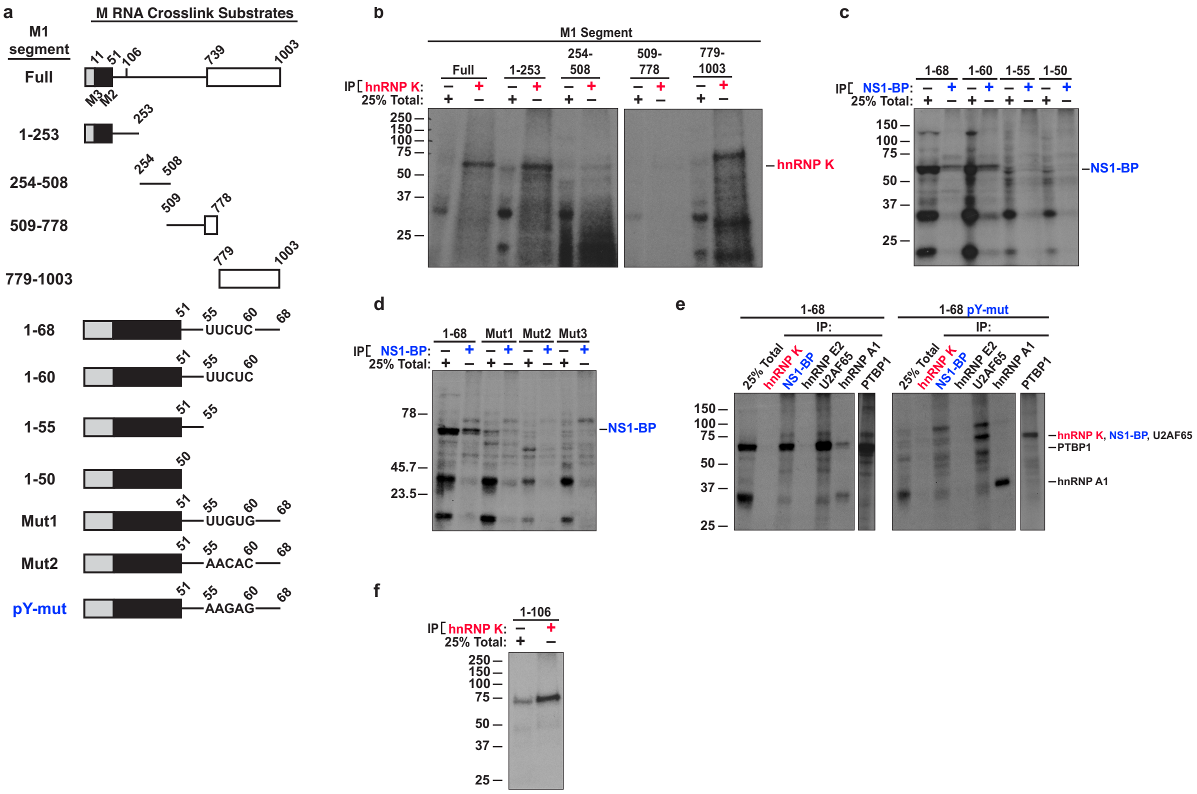

Supplementary Figure 1. PR8 M segment crosslinking using additional substrates and RNA binding protein antibodies. All crosslinking reactions were performed using uniformly radiolabeled, *in vitro* transcribed RNA probes derived from IAV M segment mRNA and JSL1 nuclear extracts. Post protein binding and UV irradiation (254 nm), crosslink reactions were RNase treated. For total lanes 25% of the reaction was loaded. For IP lanes, indicated proteins were immunoprecipitated from RNase-treated crosslink reactions via primary antibody and corresponding protein a/g beads. 100% of bead eluate was loaded. Sizes of immunoprecipitated crosslink species are indicated on the right of the gel image. (**a**) Diagrams of M segment-derived RNA substrates used for UV-crosslinking assays. Names of each probe are indicated on the left. Boxes denote exons and the lines denote introns. (**b**) UV-crosslinking of probes: Full, 1-253, 254-508, 509-778, and 779-1003. IP for hnRNP K. (**c**) UV-crosslinking of probes: 1-68, 1-60, 1-55, 1-50. IP for NS1-BP. (**d**) UV-crosslinking of probes: 1-68, Mut1, Mut2, and pY-mut. IP for NS1-BP. (**e**) UV-crosslinking of probes: 1-68 and pY-mut. IP for hnRNP K, NS1-BP, hnRNP E2, U2AF65, hnRNP A1, and PTBP1. (**f**) UV-crosslinking and immunoprecipitation of 1-106 probe with recombinant His-tagged hnRNP K in the absence of nuclear extract.

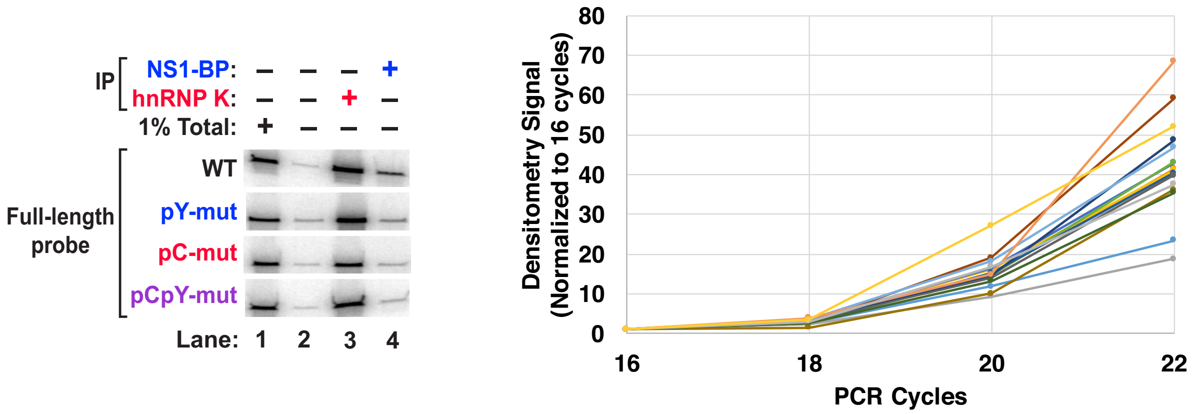

Supplementary Figure 2. Representative gel of CLIP-PCR RT-PCR products (left panel) that were quantified in **Fig. 1f**. Quantification of each RT-PCR product at different PCR cycle counts (normalized to lowest cycle number) to determine linear detection range of RT-PCR (right panel).

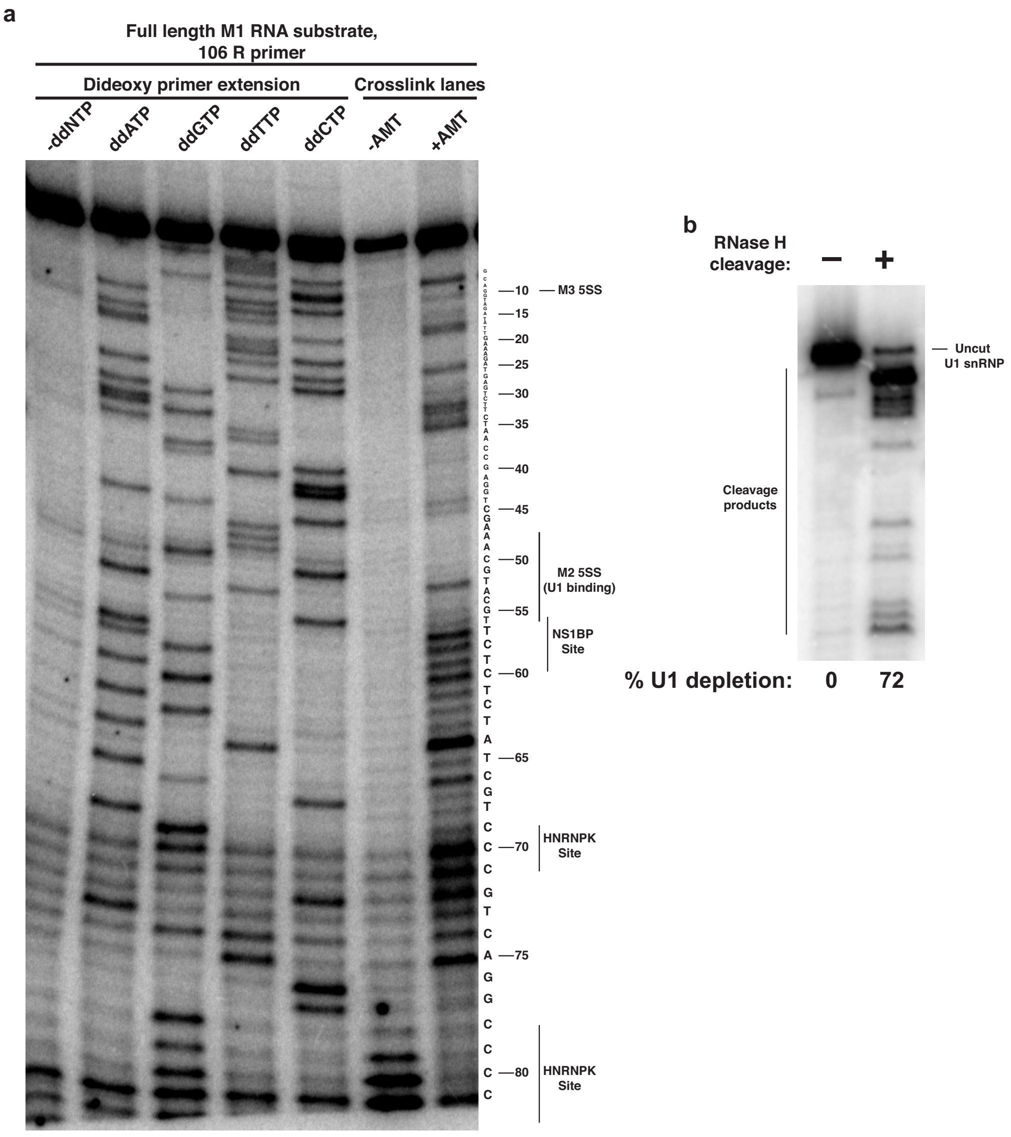

Supplementary Figure 3. (**a**) Dideoxy sequencing ladder of IAV M segment nucleotides 1-106 using a radiolabeled primer at position 106. Nucleotide identity and splicing regulatory features are indicated on right of image. (**b**) Quantification of RNase H mediated cleavage of U1 snRNA with uncleaved in first lane and cleaved in second lane.

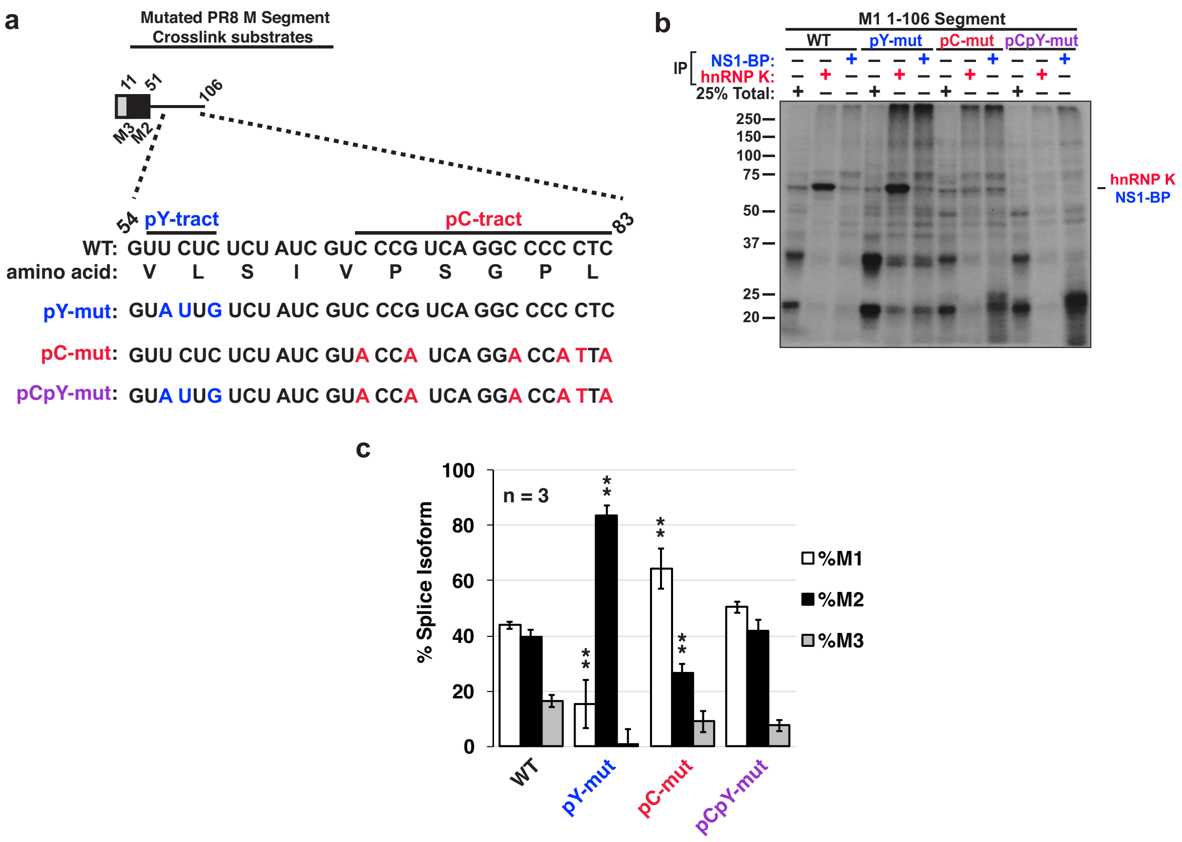

Supplementary Figure 4. UV-crosslinking of PR8 mutant M segments and quantification of PR8 mutant antisense RNA. (**a**) Diagrams of M segment mutants used in viral studies in Figures 4 and 5. Names of each probe are indicated on the left. Boxes denote exons and the lines denote introns. (**b**) UV-crosslinking of probes from panel a: WT, pY mut, pC-mut, and pCpY mut. IP for hnRNP K and NS1-BP. All crosslinking reactions were performed using uniformly radiolabeled, *in vitro* transcribed RNA probes derived from IAV M segment mRNA and JSL1 nuclear extracts. Post protein binding and UV irradiation (254 nm), crosslink reactions were RNase treated. For total lanes 25% of the reaction was loaded. For IP lanes, indicated proteins were immunoprecipitated from RNase-treated crosslink reactions via primary antibody and corresponding protein a/g beads. 100% of bead eluate was loaded. Sizes of immunoprecipitated crosslink species are indicated on the right of the gel image. (**c**) Quantification of IAV M mRNA primer extension products 12 h post IAV infection in A549 cells, moi = 2. represented as percentage signal of each isoform relative to signal of total isoforms (M1 + M2 + M3). Values are means ± s.d. from three independent experiments. Statistical significance was determined via two-tailed student's t-test where $p<0.1$ = * and $p<0.05$ = ** when comparing mock vs siRNA samples.

Fig. 1b

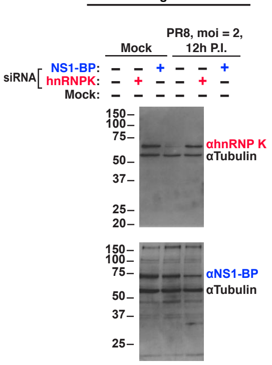

Fig. 1c

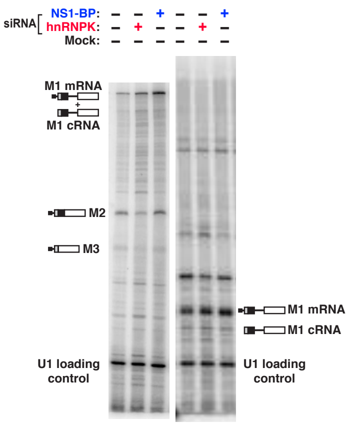

Fig. 2

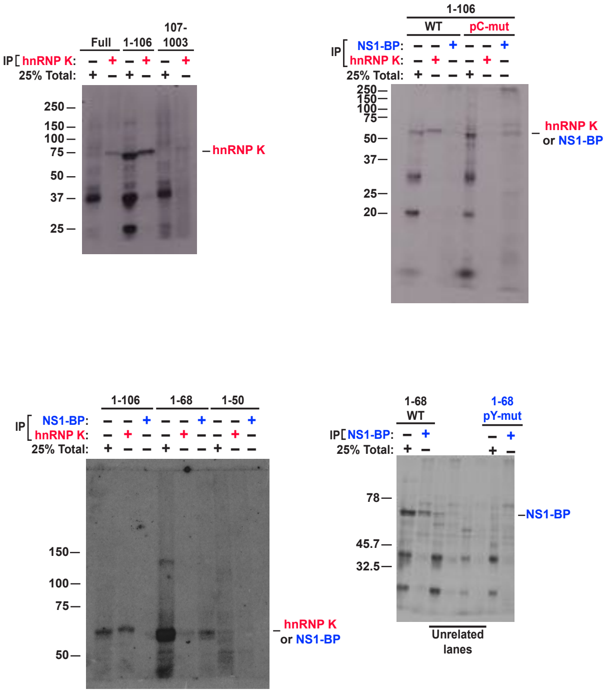

Fig. 3b

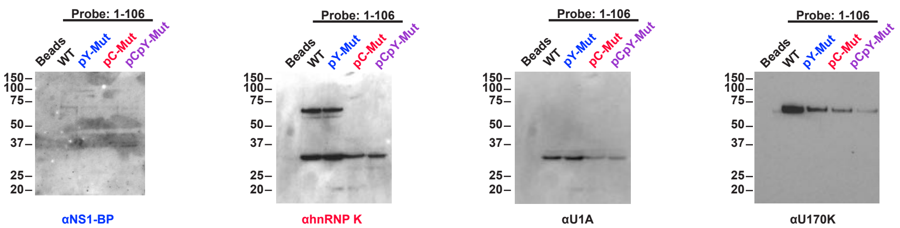

Fig. 3d

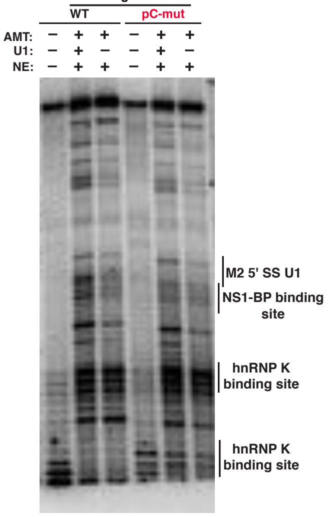

Fig. 4

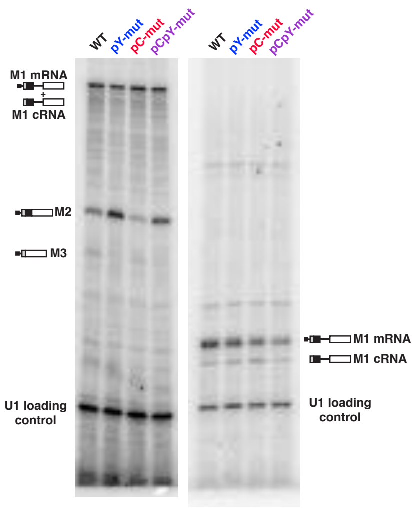

Fig. 5a

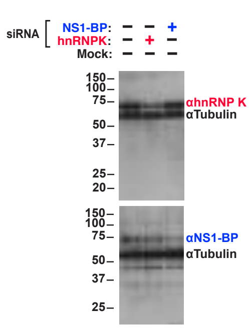

Fig. 4e

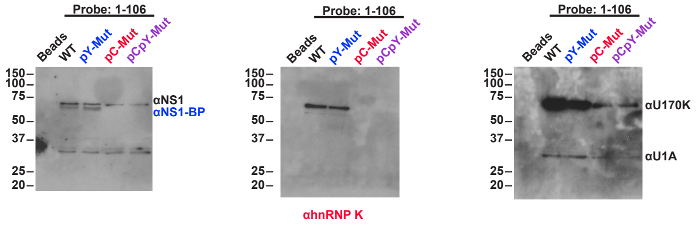

Fig. 6c

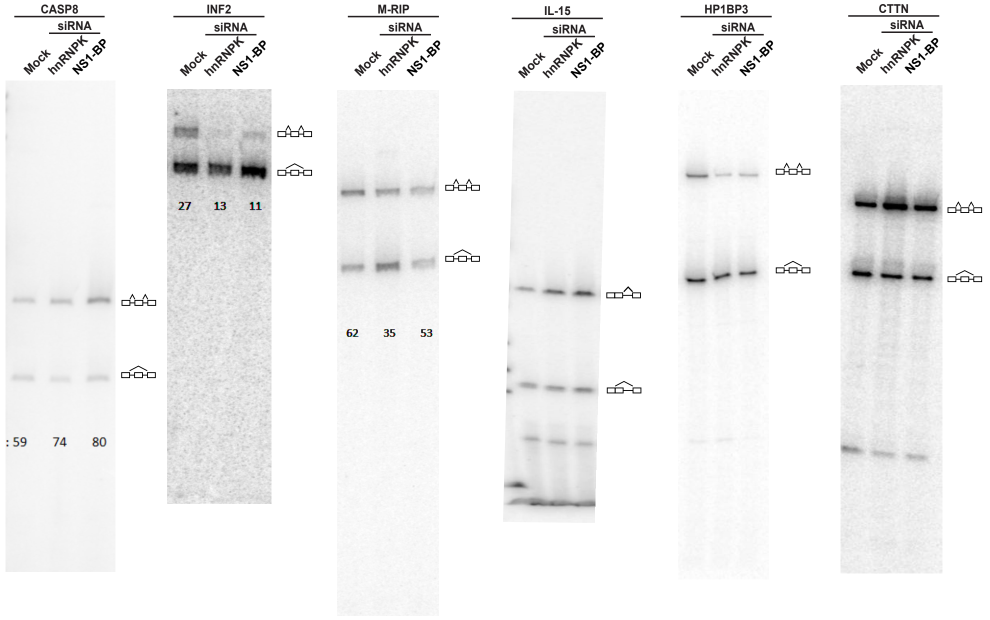

Fig. 6d

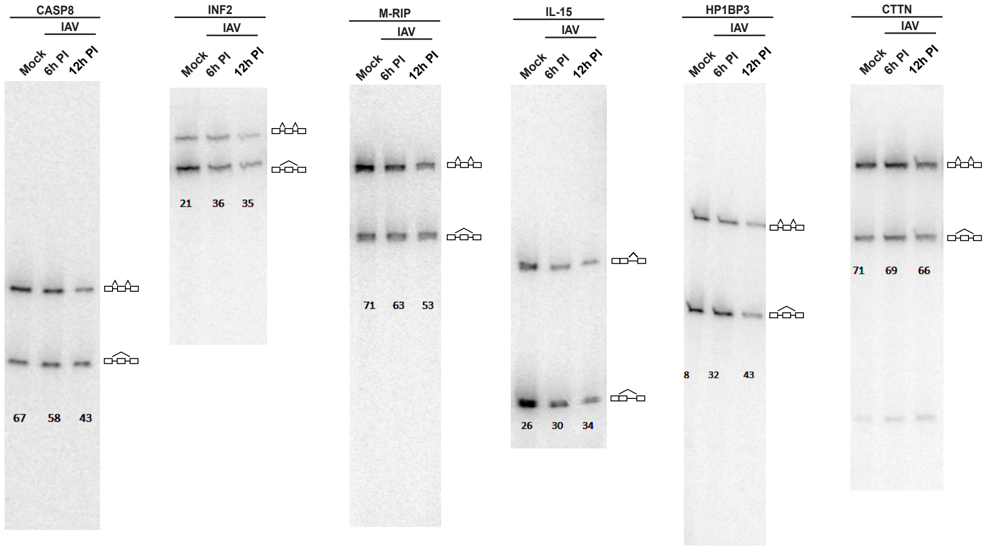

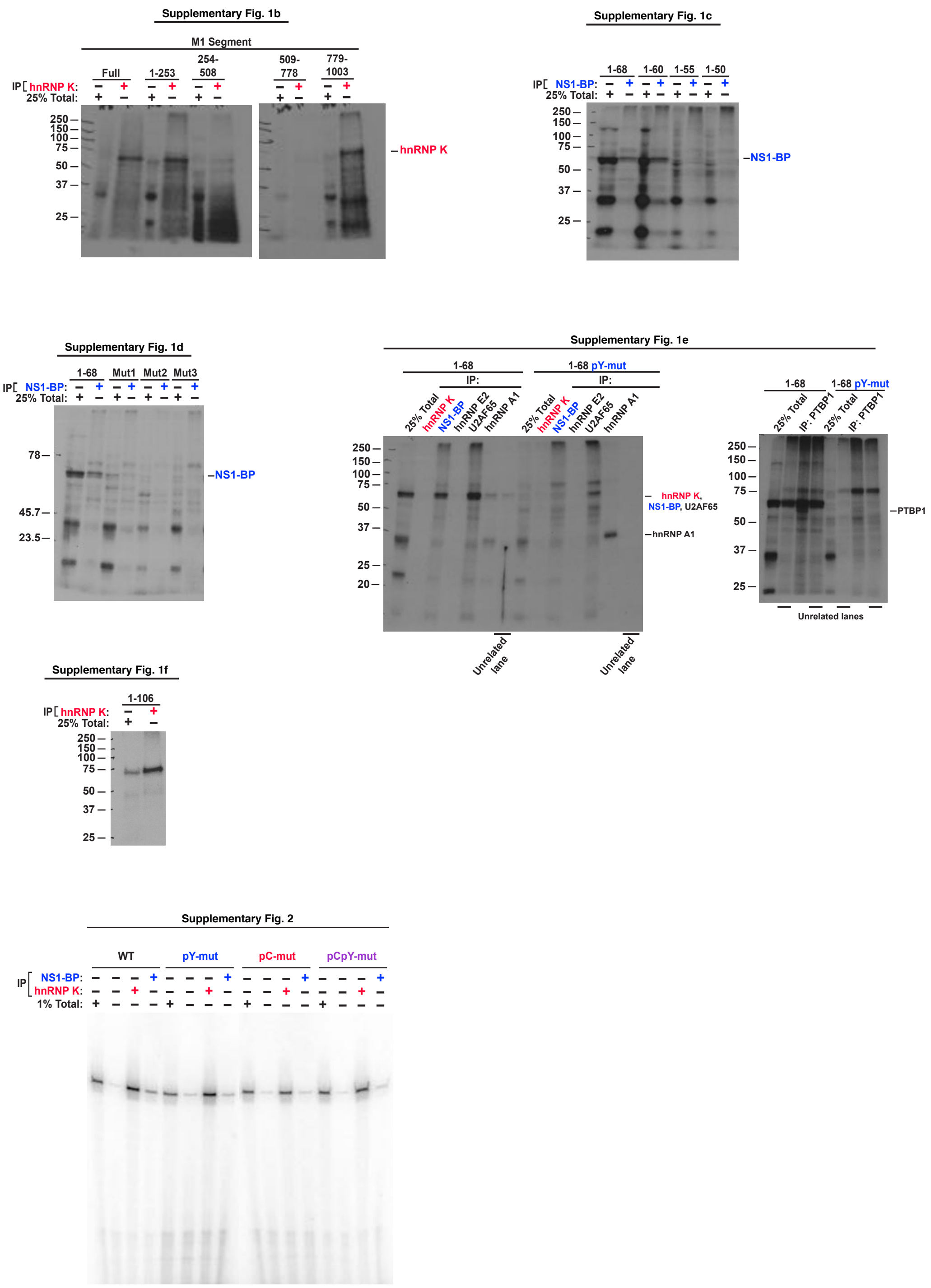

Supplementary Figure 5. Uncropped gels with corresponding cropped figure indicated above each image.

	RT-PCR Validation		RASL Predictions	
Gene Name	∆PSI sihnRNP K	∆PSI siNS1-BP	∆PSI sihnRNP K	∆PSI siNS1-BP
EIF2AK4	-24.8	-12.6	-15.5	-8.9
CASP8	29.7	28.2	24.4	27.9
DDR1	-20.7	-18.1	-20.8	-18.4
NASP	-18.8	-16.0	-32.8	-23.4
PEX5	8.4	5.4	16.4	16.2
EPB41L1	4.3	2.3	32.3	16.2
GTF2I	20.0	8.1	18.7	11.6
CARM1	16.5	13.3	20.8	18.1
NFAT5	-11.3	-12.0	-13.4	-11.2
DMN1L	30.3	17.8	18.4	14.0
HISPPD	44.5	0.0	52.4	11.0
DKFZp434I0612	-15.9	-13.5	-14.0	-10.6
C5orf33	-12.7	-9.1	-24.8	-15.5
INF2	-16.7	-14.9	-17.9	-16.1
SEC31A	9.1	0.1	-16.2	-23.5
CTTN	21.4	21.9	14.2	10.6
IL-15	15.8	13.5	12.7	16.6
MLL5	9.3	-0.7	10.5	-11.5
NUMB	13.4	-4.1	24.6	-10.4
SETD8	-6.8	-13.0	-16.9	18.0
MYOF	-44.0	-2.3	-32.4	-0.8
HNRPDL	-13.0	-1.7	-27.0	-3.4
CKLF	13.7	4.0	27.1	7.0
TACC2	25.0	6.3	21.5	5.8
SEC31A	8.2	0.7	18.6	1.8
ASAP1	41.7	6.0	20.5	6.4
M-RIP	-29.3	-12.7	-32.5	-8.3
MPRIP	5.0	14.0	4.0	17.7
MARK2	-14.0	1.0	-5.2	15.3
GIT1	-3.0	-5.0	-2.4	-11.0
POLPID3	-10.0	2.0	8.4	20.8
TJP1	10.8	-32.8	-9.1	-39.4
EFNA1	-1.3	2.0	-3.9	10.7
SMARCA1	11.3	-19.7	7.2	-19.9

Legend

(red)	hnRNP K responsive
(blue)	NS1-BP responsive
(purple)	hnRNP K/ NS1-BP responsive
(grey)	hnRNP K /NS1-BP unresponsive

Supplementary Table 1. RT-PCR validations of RASL events. All experiments represent data from three independent experiments.
