# Supplementary material for: Co-regulatory activity of hnRNP K and NS1-BP in influenza and human mRNA splicing
Source: Nat Commun. 2018 Jun 19;9:2407. doi: 10.1038/s41467-018-04779-4 (PMC6008300; doi:10.1038/s41467-018-04779-4)
Supplement: Supplementary file 2 — Description of Additional Supplementary Files [file 41467_2018_4779_MOESM2_ESM.pdf]

Description of Additional Supplementary Files

Supplementary Data 1. RASL data for events expressed in A549 cells with an average read depth of 10 across all experiments.

Supplementary Data 2. Summary of all primers used to generate RNA templates, perform primer extension, and RT-PCR validations.
